# Prognostic role of ACTL10 in Cytogenetic Normal Acute Myeloid Leukemia

**DOI:** 10.7150/jca.39467

**Published:** 2020-06-29

**Authors:** Rui Lai, Weilong Zhang, Xue He, Xinhui Liao, Xiaoni Liu, Wei Fu, Ping Yang, Jing Wang, Kai Hu, Xiaoliang Yuan, Xiuru Zhang, Hongmei Jing, Weiyou Liu

**Affiliations:** 1 Department of the Respiratory medicine, The People's Hospital of Ruijin City, Ruijin, 342500, China.; 2 Department of Hematology, Lymphoma Research Center, Peking University Third Hospital, Beijing, 100191, China.; 3 Department of Pathology, Beijing Tiantan Hospital Affiliated with Capital Medical University, No. 6 Tiantan Xili, Beijing, 100050, China.; 4 Department of Respiratory medicine, First Affiliated Hospital Gannan Medical University, Ganzhou, 341000, China.; 5 Peking University Third Hospital, Beijing, 100191, China.

**Keywords:** ACTL10, CN-AML, RNA expression, DNA methylation

## Abstract

ACTL10 is a member of the actin family; however, despite previous studies suggesting that certain proteins in this family may be related to the pathogenesis of leukemia, to the best of our knowledge, no studies to date have demonstrated any association between ACTAL10 and leukemia. Thus, the present study aimed to determine the association between ACTL10 expression levels, DNA methylation levels and the clinical prognosis in cytogenic normal acute myeloid leukemia (CN-AML). Data from seventy-five patients with CN-AML and patients with AML treated with chemotherapy or allogeneic hematopoietic stem cell transplantation were obtained from The Cancer Genome Atlas (TCGA) dataset and were used to analyze the clinical prognosis of ACTL10 RNA expression levels and DNA methylation levels. In addition, the study also investigated the combined clinical prognosis of ACTL10 RNA expression levels and ACTL10 DNA methylation levels in 74 patients with CN-AML from the TCGA dataset. ACTL10 RNA expression levels were observed to be highly expressed in patients with CD34^+^/CD38^+^ AML (P<0.01). Both ACTL10 RNA expression levels and DNA methylation were found to be independent prognostic factors for patients with CN-AML; patients with CN-AML in the ACTL10 RNA-high expression group had an increased EFS (P=0.0016) and OS (P=0.014) and patients in ACTL10 DNA methylation-low group also demonstrated a long EFS (P<0.0001) and OS (P=0.004). Notably, integrating ACTL10 RNA expression levels and ACTL10 DNA methylation levels could more accurately predict the prognosis of patients with CN-AML (EFS and OS, P<0.0001). In conclusion, the findings of the present study suggested that the high RNA expression levels and low DNA methylation levels of ACTL10 may predict a good prognosis in patients with CN-AML.

## Introduction

Acute myeloid leukemia (AML) is the most common hematological malignancy in adults, with a median age of diagnosis of 65 years old 1, 2. AML is characterized by the malignant cloning of bone marrow hematopoietic stem cells, leading to abnormal erythropoiesis and bone marrow failure 1, 2 and the main clinical manifestations of AML are anemia, hemorrhages, infections and the proliferation of leukemia cells 3. There are currently two common systems for classifying AML: the France-USA-Britain (FAB) system divides AML into 8 subtypes, M_0_ to M_7_, whilst the World Health Organization divides AML into 6 subtypes 4, 5. In recent years, improved treatment options, including potent chemotherapeutics, targeted therapies, such as FLT3 inhibitors, IDH inhibitors and Bcl-2 inhibitors, and hematopoietic stem cell transplantation (HSCT) have markedly improved the prognosis of patients with AML; however, AML is still associated with high incidence, mortality and recurrence rates. At present, 35-40% of patients with AML and ≤60 years old are cured, whereas only 5-15% of patients >60 years old are successfully treated, particularly those patients who cannot receive potent chemotherapeutic regimens, who have a median survival time of 5-10 months 6. The prognosis of patients with AML is closely related to genetic mutations and molecular markers; for example, chromosomal translocations t(8; 21), t(15; 17) or inv(16) have been reported as beneficial prognostic factors; t(9; 11), monomer 5 or 7 and normal cytogenetics are considered to be moderate risk factors; and t(6; 9), inv(3) or 11q changes have been observed to lead to treatment failure and death, so are considered as high risk factors. In addition, t(8; 21) patients with AML and C-KIT mutations have an increased risk of recurrence and reduced overall survival (OS) 2, 3.

Cytogenetic normal acute myeloid leukemia (CN-AML) is considered as a moderate risk AML based on the treatment and cytogenetic classification, and accounts for ~45% of adult AML cases and 20% of pediatric AML cases 7, 8. Previous studies have reported that the prognosis of CN-AML can be predicted by identifying mutations in certain genes; for example, NPM1 and CEBPA gene mutations are acquired mutations in CN-AML that have been found to predict a good prognosis 9-11, whereas KIT, RUNX1, IDH1, WT1, FLT3, TET2, ASXL1 and DNMT3A gene mutations are malignant mutations in CN-AML that have been reported to predict a poor prognosis 10, 12-14. Thus, there is an urgent requirement to identify more prognostic markers to better assess the prognosis of CN-AML.

Actin-like 10 (ACTL10) is a member of the actin family; however, to the best of our knowledge, there are very few studies on the ACTL10 gene. Actin-like proteins serve major roles in muscle contraction, cell growth, movement and shape determination, such as the regulation of cell proliferation, migration and differentiation 15-17. Actin-like 6 (ACTL6) is also homologous to the actin-like proteins and has an important role in embryonic development and stem cell and progenitor maintenance. ACTL6 is closely associated with neurodevelopmental (intelligence and language) disorders and a poor prognosis in numerous types of cancer, including hepatocellular carcinoma, head and neck squamous cell carcinoma, rhabdomyosarcoma, osteosarcoma and glioma 18-23. Therefore, it was hypothesized that ACTL10 function may be associated with CN-AML and the association between ACTL10 and CN-AML requires further investigations.

DNA methylation is a methyltransferase-catalyzed transfer of methyl residues to CG cytosine to form 5-methylcytosine, which promotes changes in the chromatin structure, DNA conformation, DNA stability and DNA-protein interactions to consequently control gene expression. Specific gene methylation patterns are closely associated with the prognosis of AML 24-26; for example, the methylation of TET2 and FLT3 promoted the synergy of disease alleles and multiple loci, leading to reversible leukemia transformation and thereby improving the prognosis of patients with AML 27. Therefore, the methylation of ACTL10 may also be a prognostic factor in AML that requires further investigation. Since ACTL10 is derived from the actin-like family, which regulates cell proliferation, differentiation and migration, and the actin-like family is closely related to the pathogenesis of AML, it was hypothesized that a clinical prognostic relevance may exist between ACTL10 and CN-AML. To the best of our knowledge, there is currently no research published on the ACTL10 gene. Therefore, the present study integrated data from multiple datasets of patients with CN-AML to determine the association between ACTL10 gene expression, methylation and CN-AML clinical prognosis.

## Materials and Methods

### Bioinformatics analysis

This study includes 3 datasets, namely the TCGA dataset, the GSE 76004 dataset and the GSE12417 dataset. The road map of the patients from different datasets is presented in Fig. S1.

### TCGA dataset

In this study, a total of 187 patients with AML (see Table S1) were collected from the TCGA dataset 28, including patients with normal karyotype (CN-AML) and patients with abnormal karyotype. In this study, the 187 AML patients were divided into three categories based on RNA expression and DNA methylation of ACTL10 gene, namely 130 AML patients with RNA expression, 185 AML patients with DNA methylation and 128 AML patients with both RNA expression and DNA methylation. In order to better explore the clinical prognosis of ACTL10 gene in AML patients, we divided 187 AML patients into 6 groups according to the karyotype, RNA expression of ACTL10 gene, DNA methylation of ACTL10 gene and treatment methods. In this study, these 6 groups were named C1-C6. In these 6 groups, there is overlap between the groups (see Fig. S2). The survival time (EFS and OS) of the ACTL10 RNA-high expression group and the ACTL10 RNA-low expression group were compared in the 75 patients with CN-AML (**C1**). This is the training cohort. The clinical prognosis of ACTL10 RNA-high expression group and ACTL10 RNA-low expression group were compared in 92 AML patients received chemotherapy (**C2**). The clinical prognosis of the ACTL10 DNA methylation-high group and the ACTL10 DNA methylation-low group were compared in the 85 patients with CN-AML (**C3**). The clinical prognosis of ACTL10 DNA methylation-high group and ACTL10 DNA methylation-low group were compared in 77 AML patients (**C4**) who had received allogeneic hematopoietic stem cell transplantation (Allo-HSCT). The clinical prognosis of the ACTL10 DNA methylation-high group and the ACTL10 DNA methylation-low group were compared in 101 AML patients received chemotherapy (**C5**). We also integrated ACTL10 RNA expression levels and ACTL10 DNA methylation levels from 74 CN-AMLs to better assess the prognosis of CN-AML patients (**C6**).

### GSE12417 dataset

We collected 78 CN-AML patients from the GSE12417 dataset 29 and compared the clinical prognosis groups of the ACTL10 RNA-high expression group and ACTL10 RNA-low expression group. This is the validation cohort.

### GSE76004 dataset

We collected 78 AML patients from the GSE76004 dataset 30 and compared ACTL10 RNA expression levels in patients with different leukemia stem cell (LSC) activity. The LSC activity of 227 CD34/CD38 cell fractions (138 LSC+ and 89 LSC-) from the 78 AML patients was determined using a xenotransplantation assay.

The current study was conducted in accordance with the Declaration of Helsinki and the study was approved by the Ethics Committee of Peking University Third Hospital and the Ethics Committee of Gannan Medical University. Written informed consent was obtained from all patients 28-30.

### RNA expression levels and DNA methylation analysis

The robust multiarray average method was used to calculate RNA expression microarrays in all CN-AML samples from the Gene Expression Omnibus dataset. To facilitate data calculation, log2 was used to convert the expression levels of each probe. RNA expression levels in patients from the TCGA dataset was obtained by RNA-Seq and the expression levels were presented as RPKM (millions of readings per kilogram of mapping) and then transformed by log2 (FPKM +1). The DNA methylation status of patients from the TCGA dataset was determined by human methylation of the 450k chip and the expression levels were presented as between 0 to 1 using the ACTL10 related probe; 0 indicated no methylation and 1 indicated 100% methylation.

The P-value and hazard ratio (HR) of the whole genome gene expression profile and the whole genome DNA methylation profile was calculated using the prognosis of CN-AML from the TCGA dataset and the HR function in the survcomp package. The ACTL10 gene met the criterion of P<0.05 for both RNA expression and DNA methylation. Subsequently, ACTL10 RNA expression levels were divided into a high expression group and a low expression group (ACTL10 RNA-high expression group and ACTL10 RNA-low expression group) through the maximally selected rank statistics method from the surv_cutpoint package of survminer. The methylation status of ACTL10 DNA was also divided into a methylated high group and a methylated low group (ACTL10 DNA methylation-high group and ACTL10 DNA methylation-low group) using the maximally selected rank statistics method from the surv_cutpoint package of survminer. The ACTL10 RNA expression levels and the ACTL10 DNA methylation status in 74 patients with CN-AML (TCGA dataset) were integrated and subsequently divided into four groups through the maximally selected rank statistics method using the surv_cutpoint package of survminer: i) Group 1 (G1) contained patients with high expression levels of ACTL10 RNA and a high methylation status of ACTL10 DNA; ii) group 2 (G2) contained patients with high expression levels of ACTL10 RNA, but a low methylation status of ACTL10 DNA; iii) group 3 (G3) contained patients with low expression levels of ACTL10 RNA and a high methylation status of ACTL10 DNA; and iv) group 4 (G4) contained patients with low expression levels of ACTL10 RNA and a low methylation status of ACTL10 DNA.

### Statistical analysis

Statistical analysis was performed using ggplot2 and survivor packages of R v3.1.3 software. Statistical differences between two groups were determined using a Student's t-test, whilst comparisons among >2 groups were determined using an ANOVA. The Fisher's exact test was used to analyze the enumeration data and the Log-rank test was used for survival analysis. Cox regression analysis of multivariate analysis was used to analyze the hazard ratios of various biomarkers associated with CN-AML.

## Results

### Baseline characteristics of patients with CN-AML are relatively consistent

The ACTL10 RNA-high expression group and the ACTL10 RNA-low expression group from the TCGA dataset had 52 and 23 patients with CN-AML, respectively. The ACTL10 DNA methylation-high group and the ACTL10 DNA methylation-low group from the TCGA dataset had 26 and 59 patients with CN-AML, respectively. To ensure that the contrast between the each two groups was accurate and bias was reduced, the two groups of patients were ensured to have consistent clinical characteristics, such as sex, race, FAB type, age, bone marrow blast cell, peripheral blood WBC and peripheral blood blast cell (RNA expression, P>0.05; DNA methylation, P>0.05; Fisher's exact test; Table 1). There were no statistical differences observed between the induction therapy, transplantation protocol or pre-transplant status (RNA expression, P>0.05; DNA methylation, P>0.05; Fisher's exact test; Table 1) of these patients with CN-AML.

No significant differences were observed in the relapse rate (%) between the RNA-high expression group and RNA-low expression group (P=1; Fisher's exact test; Table 1). However, the relapse rate (%) between the DNA-high expression group and DNA-low expression group was significantly different (P=0.004; Fisher's exact test; Table 1); in these patients, there was no difference in the mutations, unknown or wild-type (WT), of the DNMT3A, NPM1, TET2, FLT3, IDH2, IDH1, RUNX1, NRAS, WT1, CEBPA, PTPN11 or KRAS genes (RNA expression, P>0.05; DNA methylation, P>0.05; Fisher's exact test; Table 1).

### High RNA expression levels of ACTL10 predicts a good prognosis in patients with CN-AML

**Figure 1:**
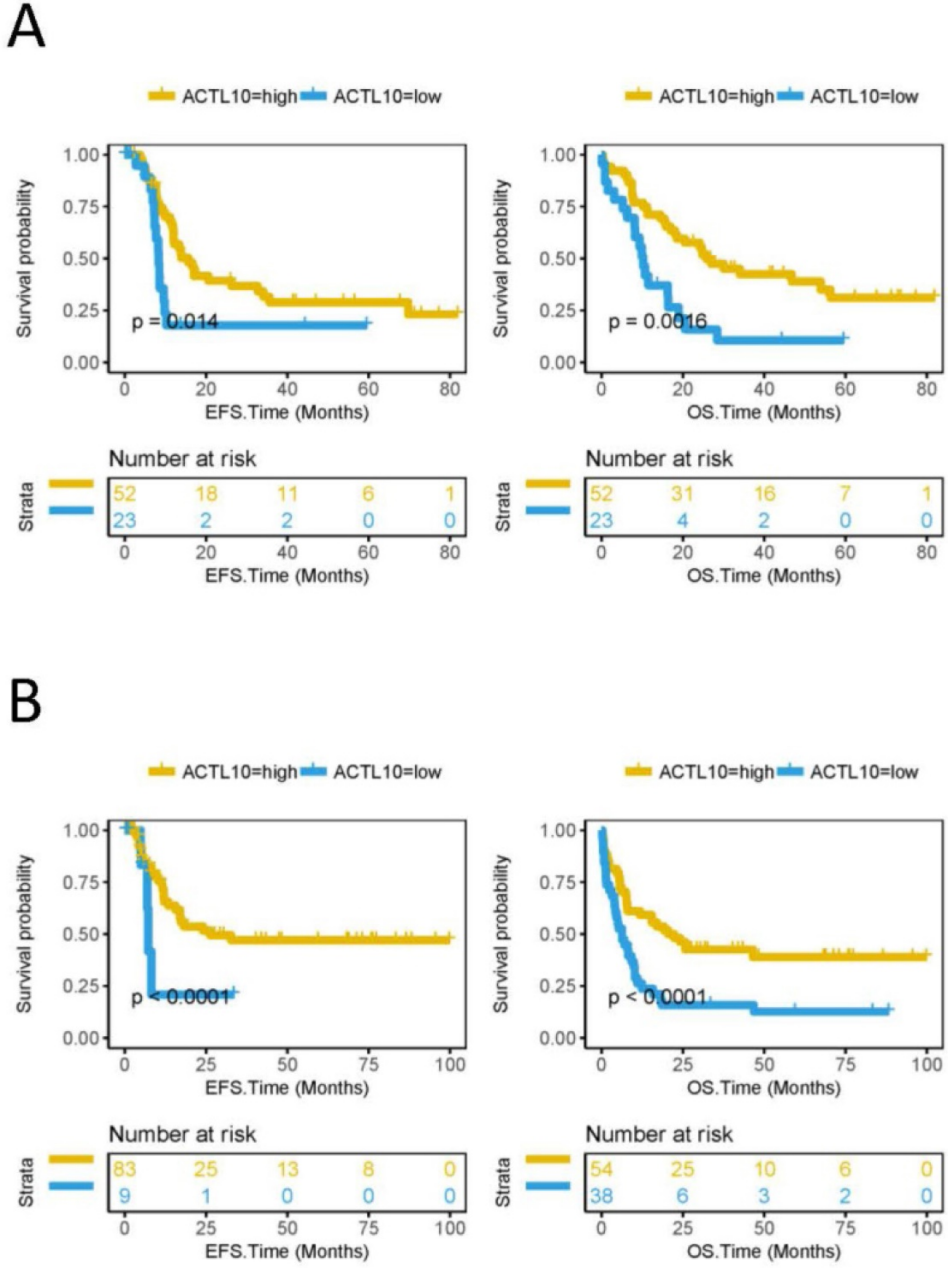
EFS and OS between the ACTL10 RNA-high expression group and the ACTL10 RNA-low expression group in patients with CN-AML. Left side: the x-axis represents the EFS time (months) and the y-axis represents the survival probability. Right side: the x-axis represents the OS time (months) and the y-axis represents the survival probability. All data was obtained from the TCGA dataset. (**A**) Data from 75 patients with CN-AML (training cohort). (**B**) Data from 92 patients with AML who received chemotherapy. Data were analyzed using a log-rank test. EFS, event-free survival time; OS, overall survival time.

The EFS and OS were compared between the ACTL10 RNA-high expression group and the ACTL10 RNA-low expression group in 75 patients with CN-AML (TCGA dataset; training cohort) and 92 patients with AML who received chemotherapy (TCGA dataset). It was observed that the ACTL10 RNA-high expression group had a longer EFS (CN-AML, P=0.014; AML chemotherapy, P<0.0001; log-rank test; Fig. 1) and OS (CN-AML, P=0.0016; AML chemotherapy, P<0.0001; log-rank test; Fig. 1). In addition, the OS in the ACTL10 RNA-high expression group and the ACTL10 RNA-low expression group in 78 patients with CN-AML from the GSE12417 dataset (validation cohort) was subsequently investigated; the result were consistent with the previous results, demonstrating that the OS of the ACTL10 RNA-high expression group was increased compared with the ACTL10 RNA-low expression group (OS, P<0.0001; log-rank test; Fig. S3).

### Expression levels of ACTL10 RNA are low in CD34^+^/CD38^-^ patients with AML

To compare the expression levels of ACTL10 RNA in hematopoietic stem cells and hematopoietic progenitor cells (CD34/CD38), 227 CD34/CD38 cell fractions from 78 patients with AML were analyzed. Decreased ACTL10 RNA expression levels were found in CD34^+^/CD38^-^ patients (dataset GSE76004; two-tailed unpaired t-test; P<0.01; Fig. S4), whereas ACTL10 RNA expression levels were observed to be increased in CD34^+^/CD38^+^ patients with AML (dataset GSE76004; two-tailed unpaired t-test; P<0.05; Fig. S4). However, the differences in the expression levels of ACTL10 RNA were not statistically significant between CD34^-^/CD38^-^ and CD34^-^/CD38^+^ patients (GSE76004; two-tailed unpaired t-test; P>0.05; Fig. S4).

### Lower DNA methylation status of ACTL10 predicts a better prognosis in patients with CN-AML

**Figure 2:**
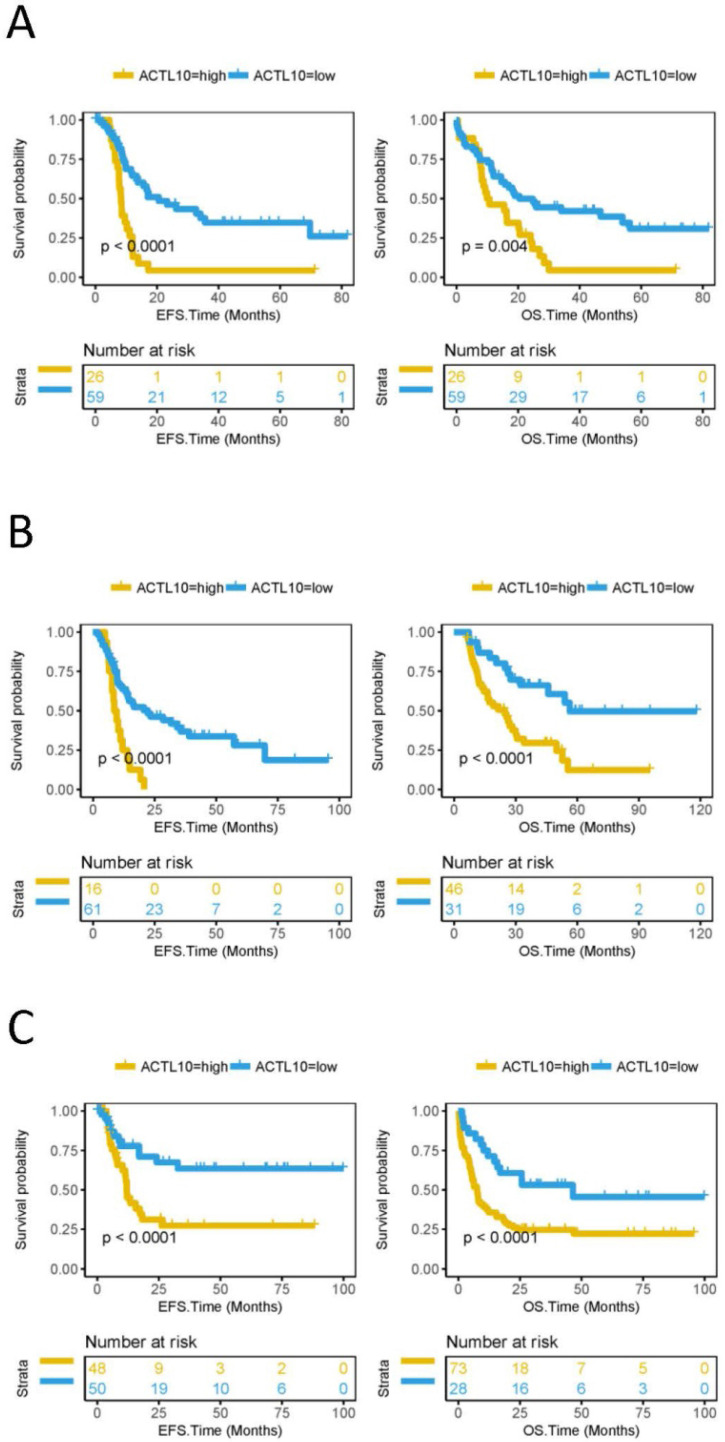
EFS and OS between the ACTL10 DNA methylation-high group and the ACTL10 DNA methylation-low group were compared in patients with CN-AML. Left side: the x-axis represents the EFS time (months) and the y-axis represents the survival probability. Right side: the x-axis represents the OS time (months) and the y-axis represents the survival probability. All data was obtained from the TCGA dataset. (**A**) Survival curve for 85 patients with CN-AML. (**B**) Survival curve for 77 patients with AML who received allogeneic hematopoietic stem cell transplantation. (**C**) Survival curve for patients with AML who received chemotherapy. Left side: EFS was analyzed in 98 patients with AML who received chemotherapy. Right side: OS was analyzed in 101 patients with AML who received chemotherapy. Data were analyzed using a log-rank test. EFS, event-free survival time; OS, overall survival time.

EFS and OS was subsequently compared between the ACTL10 DNA methylation high-group and the ACTL10 DNA methylation low-group in 85 patients with CN-AML (TCGA dataset); patients with CN-AML and low levels of ACTL10 DNA methylation had a longer EFS (P<0.0001; log-rank test; Fig. 2A) and OS (P=0.004; log-rank test; Fig. 2A). In addition, EFS and OS were also compared between the ACTL10 DNA methylation high group and the ACTL10 DNA methylation low group in patients with AML, who had received different treatment regimens (77 patients with AML who had received Allo-HSCT and 101 patients with AML who had received chemotherapy; TCGA datasets). Similarly, lower levels of ACTL10 DNA methylation were associated with a longer EFS (P<0.0001; log-rank test; Fig. 2B and C) and OS (P<0.0001; log-rank test; Fig. 2B and C) in patients with AML who had received different treatment regimens.

### Integration of ACTL10 RNA expression levels and ACTL10 DNA methylation levels can better predict survival in patients with CN-AML

**Figure 3:**
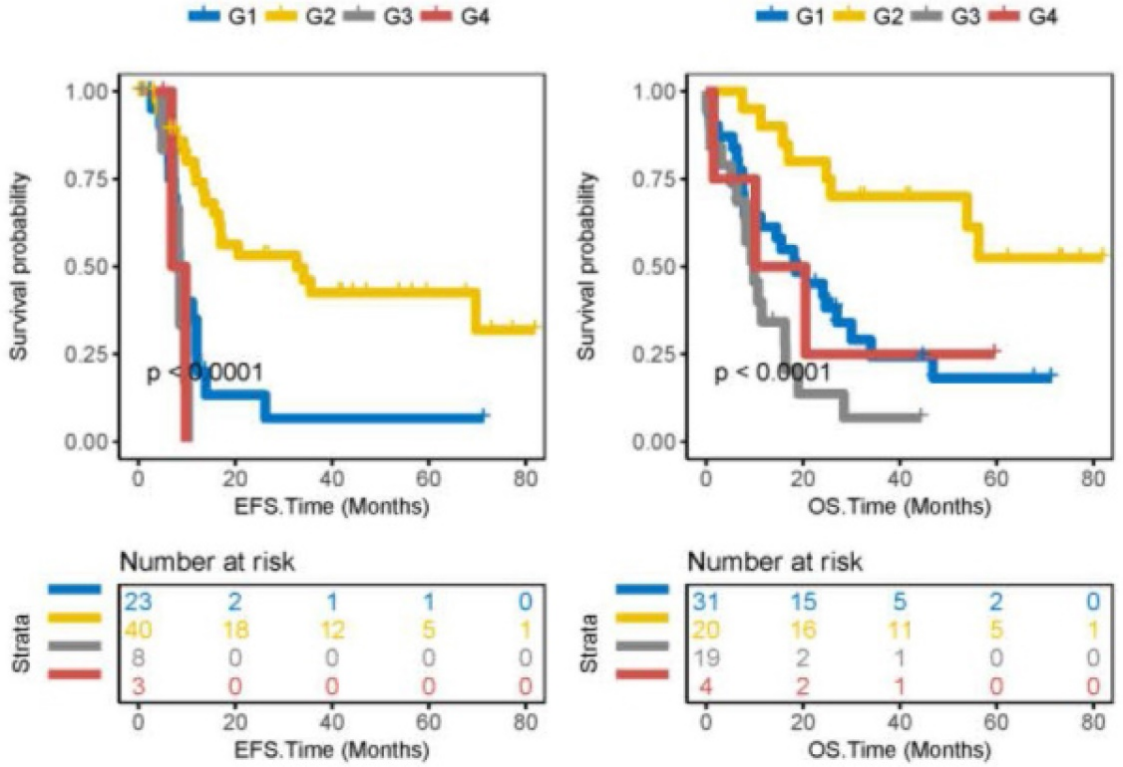
Integration of ACTL10 RNA expression levels and ACTL10 DNA methylation levels to predict survival time. Left side: the x-axis represents the EFS time (months) and the y-axis represents the survival probability. Right side: the x-axis represents the OS time (months) and the y-axis represents the survival probability. Data was analyzed from 74 patients with CN-AML from the TCGA dataset. G1 represents patients with high expression levels of ACTL10 RNA and high methylation levels of ACTL10 DNA; G2 represents patients with high expression levels of ACTL10 RNA and low methylation levels of ACTL10 DNA; G3 represents patients with low expression levels of ACTL10 RNA and high methylation levels of ACTL10 DNA; G4 represents patients with low expression levels of ACTL10 RNA and low methylation levels of ACTL10 DNA. Data were analyzed using a log-rank test. EFS, event-free survival time; OS, overall survival time.

The prognosis of 74 patients with CN-AML was predicted by integrating ACTL10 RNA expression levels and ACTL10 DNA methylation levels; ACTL10 RNA expression levels and the ACTL10 DNA methylation level were integrated in 74 patients with CN-AML (TCGA dataset), which were divided into four groups (G1-G4; see the method for detailed grouping). Patients in the G2 group were observed to have the best prognosis, moderate prognosis was witnessed in patients in the G1 and G4 groups, whilst patients in the G3 group had a poor prognosis (TCGA dataset; EFS, P<0.0001; OS, P<0.0001; log-rank test; Fig. 3).

### ACTL10 RNA expression levels and ACTL10 DNA methylation are independent prognostic factors for patients with CN-AML

The present study found that the DNA methylation level of ACTL10 was a strong independent risk factor for EFS (HR =4.6053; 95% CI, 2.1979-9.6495; P=5e-05; Cox regression analysis; Table 2) and OS (HR =3.1101; 95% CI, 1.646-5.8764; P=0.0005; Cox regression analysis; Table 2) in patients with CN-AML. In addition, the RNA expression levels of ACTL10 were found to be an independent risk factor for OS (HR =0.3975; 95% CI, 0.1868-0.8458; P=0.0166; Cox regression analysis; Table 2), however the EFS (HR =0.7691; 95% CI, 0.3454-1.7126; P=0.5204; Cox regression analysis; Table 2) was not significantly altered across the RNA expression levels of ACTL10. Age was also found to be a high risk factor for ACTL10 RNA expression (EFS, HR =2.5725; 95% CI, 1.1378-5.8163; P=0.0232; OS, HR = 3.2294; 95% CI, 1.5521-6.7192; P=0.0017; Cox regression analysis; Table 2) and ACTL10 DNA methylation (EFS, HR=3.0966; 95% CI, 1.3541-7.0816; P=0.0074; OS, HR=4.0511; 95% CI, 1.933-8.4901; P=0.0002; Cox regression analysis; Table 2) in patients with CN-AML.

Mutations in certain genes were also revealed to be relatively high risk factors for patients with ACTL10 RNA expression, such as the DNMT3A mutation (EFS, HR =2.736; 95% CI, 1.2195-6.1382; P=0.0146; Cox regression analysis; Table 2), FLT3 mutation (EFS, HR =2.9251; 95% CI, 1.1391-7.5116; P=0.0257; Cox regression analysis; Table 2) and KRAS mutation (EFS, HR =0.2035; 95% CI, 0.0503-0.8227; P=0.0255; Cox regression analysis; Table 2). Furthermore, mutations in specific genes were also found to be relatively high risk factors for patients with ACTL10 DNA methylation, including the DNMT3A mutation (EFS, HR =2.2837; 95% CI, 1.1137-4.6825; P=0.0242; Cox regression analysis; Table 2), TET2 mutation (OS, HR =0.339; 95% CI, 0.1193-0.9632; P=0.0423; Cox regression analysis; Table 2), IDH2 mutation (OS, HR =0.2552; 95% CI, 0.0843-0.7723; P=0.0156; Cox regression analysis; Table 2) and NRAS mutation (EFS, HR =0.2035; 95% CI, 0.0503-0.8227; P=0.0255; Cox regression analysis; Table 2). However, the hazard ratios of bone marrow blasts, peripheral blood WBCs, peripheral blood cells and other genetic mutations (NPM1, IDH1, RUNX1, WT1, CEBPA and PTPN11) were not statistically different compared with ACTL10 RNA expression levels or the ACTL10 DNA methylation status (Table 2).

## Discussion

Cytogenetic research in CN-AML is an increasingly popular field; mutations or the methylation status of some genes has not only been found to predict the prognosis of patients with CN-AML, but also serve as an important target for treatments 31; for example, mutations in NPM1, IDH2 and CEBPA genes have been associated with a good prognosis in CN-AM 31, 32, whereas FLT3-ITD positive, IDH1, TET2, KRAS, U2AF1 and PTPN11 mutations all predicted a poorer prognosis in AML 33-35. Notably, NRAS, SMC1A, SMC3 and RAD21 mutations have been reported to have no effect on the prognosis of patients with AML 36, 37 In addition, the methylation of TET2 and FLT3 improved the prognosis of patients with AML 27.

ACTL10 is a member of the actin family, which is closely associated with the pathogenesis of leukemia through regulating proliferation, differentiation and the migration of hematopoietic stem/hematopoietic progenitor cells; however, the clinical association between ACTL10 and CN-AML remains unknown. Thus, the present study aimed to investigate the clinical significance of the association between ACTL10 and CN-AML. At present, a large number of studies have found that the gene expression levels and DNA methylation status can better classify AML and predict the prognosis of AML 38-40. Thus, the present study not only analyzed the effects of ACTL10 expression and DNA methylation on the prognosis of patients with CN-AML, but also analyzed the effects of integrating ACTL10 expression and ACTL10 DNA methylation levels to predict the prognosis of patients with CN-AML. Upon integrating data from patients with CN-AML obtained from the three datasets using survival analysis and multivariate analysis, it was found that both RNA expression levels and the DNA methylation status of ACTL10 could predict the prognosis of patients with CN-AML.

The clinical characteristics of patients with ACTL10 RNA expression and ACTL10 DNA methylation were relatively consistent, which can rule out the interference of these factors to our results (Table 1).

In addition, the study compared EFS and OS in the ACTL10 RNA-high expression group and the ACTL10 RNA-low expression group of 75 patients with CN-AML (TCGA dataset, training cohort) and 92 patients with AML, who were treated with chemotherapy (TCGA dataset); it was observed that patients in the ACTL10 RNA-high expression group had significantly improved survival times compared with the ACTL10 RNA-low expression group, with the results from patients with different patient status in the same dataset being consistent, indicating that ACTL10 RNA expression levels may predict the prognosis of patients with CN-AML. To further validate this result, the OS of an ACTL10 RNA-high expression group and the ACTL10 RNA-low expression group in 78 patients with CN-AML from another dataset (GSE12417, validation cohort) was also compared, and the results were consistent with the previous results.

Previous studies have demonstrated that CD34^+^/CD38^-^ (hematopoietic stem cell/hematopoietic progenitor surface markers) patients with AML were refractory and patients with CD34^+^/CD38^+^ AML had a good prognosis 41-43.

The present study found that ACTL10 RNA expression levels were increased in patients with CD34^+^/CD38^+^ CN-AML, whereas ACTL10 RNA expression levels were decreased in patients with CD34^+^/CD38^-^ CN-AML. These findings further suggested that patients in the ACTL10 RNA-high expression group may have an improved prognosis compared with patients in the ACTL10 RNA-low expression group.

DNA methylation is also an important factor that has been observed to affect prognosis. Therefore, the present study compared the EFS and OS of the ACTL10 DNA methylation-high group and the ACTL10 DNA methylation-low group. Patients in the ACTL10 DNA methylation-low group had significantly increased survival rates compared with the ACTL10 DNA methylation-high group, regardless of whether the patients received chemotherapy or allogeneic hematopoietic stem cell transplantation. These findings also suggested that the ACTL10 DNA methylation status may predict the prognosis of patients with CN-AML.

To more accurately predict prognosis in patients with CN-AML, ACTL10 RNA expression levels and ACTL10 DNA methylation levels were combined in 74 patients with CN-AML; the results were found to be consistent with the previous results. Patients with high RNA expression levels of ACTL10 and low methylated ACTL10 DNA (G2) were observed to have the best prognosis. The difference in EFS between patients in the G3 and G4 group was small, which was most likely due to the small number of patients in the two groups, only 8 and 3 respectively. In addition, the prognosis of G3 patients was worse compared with G4 patients, as seen in the OS curve of G3 and G4 patients; however, due to the small number of G4 patients, this result requires further investigations before conclusions can be made.

Thus, the present study determined that both ACTL10 RNA expression levels and the ACTL10 DNA methylation status were independent risk factors for predicting the prognosis of patients with CN-AML using multivariate analysis (Table 2). In addition, patient age and several genes were also reported to be important factors in influencing the prognosis of CN-AML, which is consistent with other studies and further validates our findings.

Nonetheless, the present study has limitations and future research should focus on investigating the molecular mechanisms and conducting prospective studies on the treatment of patients with CN-AML with high ACTL10 expression levels.

In conclusion, high RNA expression levels of ACTL10 and a low DNA methylation status of ACTL10 was found to predict an improved prognosis in patients with CN-AML, irrespective of whether the patient received chemotherapy or allogeneic hematopoietic stem cell transplantation. Notably, the integration of ACTL10 RNA expression levels with ACTL10 DNA methylation levels was demonstrated to predict the prognosis of patients with CN-AML more accurately.

## Supplementary Material

Supplementary figures and tables.

## Figures and Tables

**Table 1:** Baseline patient characteristics according to the expression and methylation level of ACTL10

		Expression			Methylation		
Characteristics	Level	ACTL10 -low	ACTL10 -high	P-value	ACTL10 -low	ACTL10 -high	P-value
n		23	52		59	26	
Sex (%)	Female	15 (65.2)	23 (44.2)	0.133	26 (44.1)	14 (53.8)	0.482
	Male	8 (34.8)	29 (55.8)		33 (55.9)	12 (46.2)	
Race (%)	Black	1 (4.3)	2 (3.8)	0.374	2 (3.4)	0 (0.0)	0.706
	Others	1 (4.3)	0 (0.0)		1 (1.7)	0 (0.0)	
	Unknow	5 (21.7)	8 (15.4)		11 (18.6)	3 (11.5)	
	White	16 (69.6)	42 (80.8)		45 (76.3)	23 (88.5)	
FAB (%)	M0	1 (4.3)	2 (3.8)	0.083	5 (8.5)	1 (3.8)	0.564
	M1	6 (26.1)	18 (34.6)		14 (23.7)	10 (38.5)	
	M2	10 (43.5)	9 (17.3)		16 (27.1)	5 (19.2)	
	M4	4 (17.4)	11 (21.2)		13 (22.0)	6 (23.1)	
	M5	1 (4.3)	11 (21.2)		10 (16.9)	3 (11.5)	
	M7	1 (4.3)	0 (0.0)		0 (0.0)	1 (3.8)	
	Unknow	0 (0.0)	1 (1.9)		1 (1.7)	0 (0.0)	
Age (mean (sd))		55.65 (18.15)	54.08 (16.71)	0.715	55.46 (18.15)	55.50 (15.12)	0.992
BM_BLAST (mean (sd))		65.70 (16.21)	72.96 (18.95)	0.115	70.20 (17.64)	71.08 (19.43)	0.839
WBC (mean (sd)) × 10^9^/L		54.30 (59.65)	49.70 (54.83)	0.745	46.96 (55.21)	63.55 (71.43)	0.248
PB_BLAST (mean (sd))		52.52 (27.86)	41.10 (35.18)	0.173	40.10 (32.78)	46.73 (35.38)	0.406
Karyotype (%)	Normal	23 (100.0)	52 (100.0)	NA	59 (100.0)	26 (100.0)	NA
Risk (%)	Intermediate	23 (100.0)	52 (100.0)	NA	59 (100.0)	26 (100.0)	NA
Induction (%)	7+3	7 (30.4)	24 (46.2)	0.232	23 (39.0)	9 (34.6)	0.602
	7+3+3	5 (21.7)	14 (26.9)		12 (20.3)	8 (30.8)	
	Others	11 (47.8)	14 ( 26.9)		24 (40.7)	9 (34.6)	
Transplant (%)	Auto	1 (4.3)	3 (5.8)	0.531	2 (3.4)	3 (11.5)	0.763
	Chemotherapy	10 (43.5)	27 (51.9)		29 (49.2)	13 (50.0)	
	Haplo	0 (0.0)	1 (1.9)		1 (1.7)	0 (0.0)	
	MUD	7 (30.4)	9 (17.3)		13 (22.0)	5 (19.2)	
	no treatment	1 (4.3)	0 (0.0)		2 (3.4)	0 (0.0)	
	sib Allo	4 (17.4)	12 (23.1)		12 (20.3)	5 (19.2)	
Before_transplant (%)	CR 1	4 (17.4)	14 (26.9)	0.241	13 (22.0)	6 (23.1)	0.934
	CR 2	3 (13.0)	4 (7.7)		4 (6.8)	3 (11.5)	
	CR 3	0 (0.0)	1 (1.9)		1 (1.7)	0 (0.0)	
	No transplant	10 (43.5)	27 (51.9)		29 (49.2)	13 (50.0)	
	no treatment	1 (4.3)	0 (0.0)		2 (3.4)	0 (0.0)	
	Others	5 (21.7)	3 (5.8)		7 (11.9)	2 (7.7)	
	Rel 1	0 (0.0)	2 (3.8)		2 (3.4)	2 (7.7)	
	Rel 2	0 (0.0)	1 (1.9)		1 (1.7)	0 (0.0)	
Relapse (%)	No	9 (39.1)	19 (36.5)	1	29 (49.2)	4 (15.4)	0.004
	Yes	14 (60.9)	33 (63.5)		30 (50.8)	22 (84.6)	
DNMT3A (%)	Mutation	12 (52.2)	17 (32.7)	0.228	23 (39.0)	10 (38.5)	1
	Unknow	0 (0.0)	1 (1.9)		1 (1.7)	0 (0.0)	
	WT	11 (47.8)	34 (65.4)		35 (59.3)	16 (61.5)	
NPM1 (%)	Mutation	10 (43.5)	33 (63.5)	0.149	32 (54.2)	15 (57.7)	1
	Unknow	0 (0.0)	1 (1.9)		1 (1.7)	0 (0.0)	
	WT	13 (56.5)	18 (34.6)		26 (44.1)	11 (42.3)	
TET2 (%)	Mutation	2 (8.7)	7 (13.5)	0.8	8 (13.6)	2 (7.7)	0.803
	Unknow	0 (0.0)	1 (1.9)		1 (1.7)	0 (0.0)	
	WT	21 (91.3)	44 (84.6)		50 (84.7)	24 (92.3)	
FLT3 (%)	Mutation	12 (52.2)	18 (34.6)	0.33	21 (35.6)	12 (46.2)	0.633
	Unknow	0 (0.0)	1 (1.9)		1 (1.7)	0 (0.0)	
	WT	11 (47.8)	33 (63.5)		37 (62.7)	14 (53.8)	
IDH2 (%)	Mutation	3 (13.0)	7 (13.5)	1	7 (11.9)	4 (15.4)	0.814
	Unknow	0 (0.0)	1 (1.9)		1 (1.7)	0 (0.0)	
	WT	20 (87.0)	44 (84.6)		51 (86.4)	22 (84.6)	
IDH1 (%)	Mutation	2 (8.7)	7 (13.5)	0.8	9 (15.3)	1 (3.8)	0.258
	Unknow	0 (0.0)	1 (1.9)		1 (1.7)	0 (0.0)	
	WT	21 (91.3)	44 (84.6)		49 (83.1)	25 (96.2)	
RUNX1 (%)	Mutation	3 (13.0)	4 (7.7)	0.771	7 (11.9)	1 (3.8)	0.601
	Unknow	0 (0.0)	1 (1.9)		1 (1.7)	0 (0.0)	
	WT	20 (87.0)	47 (90.4)		51 (86.4)	25 (96.2)	
NRAS (%)	Mutation	1 (4.3)	5 (9.6)	0.763	5 (8.5)	3 (11.5)	0.791
	Unknow	0 (0.0)	1 (1.9)		1 (1.7)	0 (0.0)	
	WT	22 (95.7)	46 (88.5)		53 (89.8)	23 (88.5)	
WT1 (%)	Mutation	3 (13.0)	2 (3.8)	0.311	3 (5.1)	3 (11.5)	0.561
	Unknow	0 (0.0)	1 (1.9)		1 (1.7)	0 (0.0)	
	WT	20 (87.0)	49 (94.2)		55 (93.2)	23 (88.5)	
CEBPA (%)	Mutation	5 (21.7)	3 (5.8)	0.097	3 (5.1)	5 (19.2)	0.1
	Unknow	0 (0.0)	1 (1.9)		1 (1.7)	0 (0.0)	
	WT	18 (78.3)	48 (92.3)		55 (93.2)	21 (80.8)	
PTPN11 (%)	Mutation	2 (8.7)	3 (5.8)	0.753	4 (6.8)	2 (7.7)	1
	Unknow	0 (0.0)	1 (1.9)		1 (1.7)	0 (0.0)	
	WT	21 (91.3)	48 (92.3)		54 (91.5)	24 (92.3)	
KRAS (%)	Mutation	1 (4.3)	2 (3.8)	1	1 (1.7)	2 (7.7)	0.462
	Unknow	0 (0.0)	1 (1.9)		1 (1.7)	0 (0.0)	
	WT	22 (95.7)	49 (94.2)		57 (96.6)	24 (92.3)	

n: number of patients; BM_BLAST: Percentage of bone marrow blast cell; WBC: White blood cell of peripheral blood; PB_BLAST: Percentage of peripheral blood blast cell; TARA: All-trans retinoic acid; MUD: matched unrelated donor; sib-allo HSCT: Blood-related allogeneic gene transplantation; Haplo: haploidentical; CR 1: First complete relief; Rel 1: First relapse; WT: wild type. The t-test (comparison between the two groups) was used to analyze the measurement data. The Fisher's exact test was used to analyze the enumeration data. Log-rank test was used for survival analysis.

**Table 2:** Multivariate analysis for EFS and OS

	Expression								Methylation							
Variables	EFS				OS				EFS				OS			
	HR	Lower 95%	Upper 95%	P-value	HR	Lower 95%	Upper 95%	P-value	HR	Lower 95%	Upper 95%	P-value	HR	Lower 95%	Upper 95%	P-value
Age (≥ 60 vs < 60 years)	2.5725	1.1378	5.8163	0.0232	3.2294	1.5521	6.7192	0.0017	3.0966	1.3541	7.0816	0.0074	4.0511	1.933	8.4901	0.0002
BM_BLAST (≥ 70% vs < 70%)	0.681	0.2939	1.5779	0.3702	1.3341	0.6107	2.9143	0.4697	0.7641	0.3383	1.7261	0.5176	1.0605	0.5395	2.0847	0.8647
WBC (≥ 30 vs < 30 × 10^9^/L)	1.231	0.5412	2.8001	0.6202	1.1855	0.5588	2.5154	0.6574	1.4907	0.6326	3.5131	0.3613	1.3226	0.6549	2.6713	0.4356
PB_BLAST (≥ 50% vs < 50%)	2.0875	0.8734	4.9891	0.0978	1.5356	0.6743	3.4974	0.307	1.5147	0.649	3.535	0.337	1.3654	0.6393	2.916	0.4212
DNMT3A (Mutation vs WT)	2.736	1.2195	6.1382	0.0146	1.8003	0.8665	3.7407	0.1151	2.2837	1.1137	4.6825	0.0242	1.71	0.9114	3.2081	0.0947
NPM1 (Mutation vs WT)	0.5736	0.2174	1.5137	0.2616	1.3132	0.5354	3.2207	0.5517	0.599	0.2089	1.7176	0.3403	0.7929	0.3359	1.8717	0.5964
TET2 (Mutation vs WT)	0.6495	0.24	1.7582	0.3957	0.3256	0.1025	1.0338	0.057	0.68	0.2459	1.8803	0.4574	0.339	0.1193	0.9632	0.0423
FLT3 (Mutation vs WT)	2.9251	1.1391	7.5116	0.0257	0.6255	0.2628	1.4886	0.2888	1.7359	0.6918	4.3555	0.24	0.8241	0.3677	1.8468	0.6384
IDH2 (Mutation vs WT)	0.5593	0.1634	1.915	0.3548	0.3661	0.1187	1.1294	0.0804	0.4727	0.1293	1.7287	0.2574	0.2552	0.0843	0.7723	0.0156
IDH1 (Mutation vs WT)	0.9	0.2315	3.4991	0.8792	0.3561	0.0934	1.3581	0.1306	1.3305	0.3742	4.731	0.6591	0.6212	0.1822	2.1174	0.4467
RUNX1 (Mutation vs WT)	2.1777	0.5549	8.5461	0.2646	2.1675	0.5606	8.3811	0.2622	2.8638	0.754	10.877	0.1223	2.4935	0.7737	8.0359	0.126
NRAS (Mutation vs WT)	0.5041	0.1199	2.12	0.35	0.2164	0.0429	1.0927	0.0639	0.2035	0.0503	0.8227	0.0255	0.3544	0.1113	1.1291	0.0793
WT1 (Mutation vs WT)	1.361	0.3113	5.951	0.6822	0.7724	0.1898	3.1428	0.7183	1.5361	0.4222	5.5886	0.5147	0.8738	0.2811	2.7162	0.8157
CEBPA (Mutation vs WT)	1.2394	0.3529	4.3533	0.7378	0.8825	0.2661	2.927	0.8381	1.1389	0.333	3.895	0.8357	0.6804	0.2248	2.0592	0.4956
PTPN11 (Mutation vs WT)	1.4844	0.3461	6.3668	0.595	2.1746	0.6486	7.2903	0.2082	1.6253	0.4457	5.9276	0.4619	2.2243	0.7319	6.7602	0.1587
KRAS (Mutation vs WT)	5.1838	1.0516	25.552	0.0432	1.4234	0.3101	6.5341	0.6498	2.0753	0.3629	11.866	0.4118	1.1431	0.2516	5.1927	0.8625
ACTL10 (High vs Low)	0.7691	0.3454	1.7126	0.5204	0.3975	0.1868	0.8458	0.0166	4.6053	2.1979	9.6495	5E-05	3.1101	1.646	5.8764	0.0005

BM_BLAST: Percentage of bone marrow blast cell; WBC: White blood cell of peripheral blood; PB_BLAST: Percentage of peripheral blood blast cell; Cox regression analysis.
