# Supplementary material for: Prognostic role of ACTL10 in Cytogenetic Normal Acute Myeloid Leukemia
Source: J Cancer. 2020 Jun 29;11(17):5150–61. doi: 10.7150/jca.39467 (PMC7378917; doi:10.7150/jca.39467)
Supplement: Supplementary file 1 — Supplementary figures and tables. [file jcav11p5150s1.pdf]

Supplementary figure legends

Figure S1. Road map of the patients from different datasets. A total of 187 AML patients were collected from the TCGA dataset. C1, C2, C3, C4, C5 and C6 are the codes of the six comparison groups that we defined manually. C1: 75 CN-AML patients who compared survival time (EFS and OS) between ACTL10 RNA-high expression group and ACTL10 RNA-low expression group. C2: 92 AML patients received chemotherapy who compared clinical prognosis between ACTL10 RNA-high expression group and ACTL10 RNA-low expression group. C3: 85 CN-AML patients who compared clinical prognosis between ACTL10 DNA methylation-high group and the ACTL10 DNA methylation-low group. C4: 77 AML patients received Allo-HSCT who compared clinical prognosis between ACTL10 DNA methylation-high group and the ACTL10 DNA methylation-low group. C5: 101 AML patients received chemotherapy who compared clinical prognosis between ACTL10 DNA methylation-high group and the ACTL10 DNA methylation-low group. C6: 74 CN-AML who integrated ACTL10 RNA expression data with ACTL10 DNA methylation data. The patients in these 6 groups (C1-C6) are partially overlapping. For details, see Figure S2.

Figure S2. Venn diagram of patients in each group. A: Venn diagram of patients with ACTL10 RNA expression. In the C1 and C2 groups, 37 AML patients were overlapping. B: Wayne diagram of patients with ACTL10 DNA methylation. In the C3 and C4 groups, 36 AML patients were overlapping; In the C3 and C5 groups, 42 AML patients were overlapping; Patients in the C4 and C5 groups did not overlap. C: Venn diagram of CN-AML patients. In the C1 and C3 groups, 74 CN-AML patients were overlapping. Patients with overlapping C1 and C3 are patients with C6. D: The proportion of CN-AML patients in all patients.

Figure S3. OS between the ACTL10 RNA-high expression group and the ACTL10 RNA-low expression group were compared in patients with CN-AML. The x-axis represents the OS time (days) and the y-axis represents the survival probability. Data were analyzed in 78 patients with CN-AML from the GSE12417 dataset (validation cohort) using a log-rank test. OS, overall survival time.

Figure S4. RNA expression levels of ACTL10 in $CD34^-/CD38^-$, $CD34^+/CD38^-$, $CD34^-/CD38^+$ and $CD34^+/CD38^+$ cells from patients with CN-AML. The RNA expression levels of ACTL10 are converted to log2; each dot represents each CD34/CD38 cell of a total of 227 CD34/CD38 cells, 138 leukemia stem cell positive (LSC+) cells and 89 leukemia stem cell negative (LSC-) cells. The average values of two samples were compared using an unpaired t-test, whereas the average values of >2 samples were compared using an ANOVA. NS, P>0.05, *P<0.05, **P<0.01.

Supplementary Figure 1

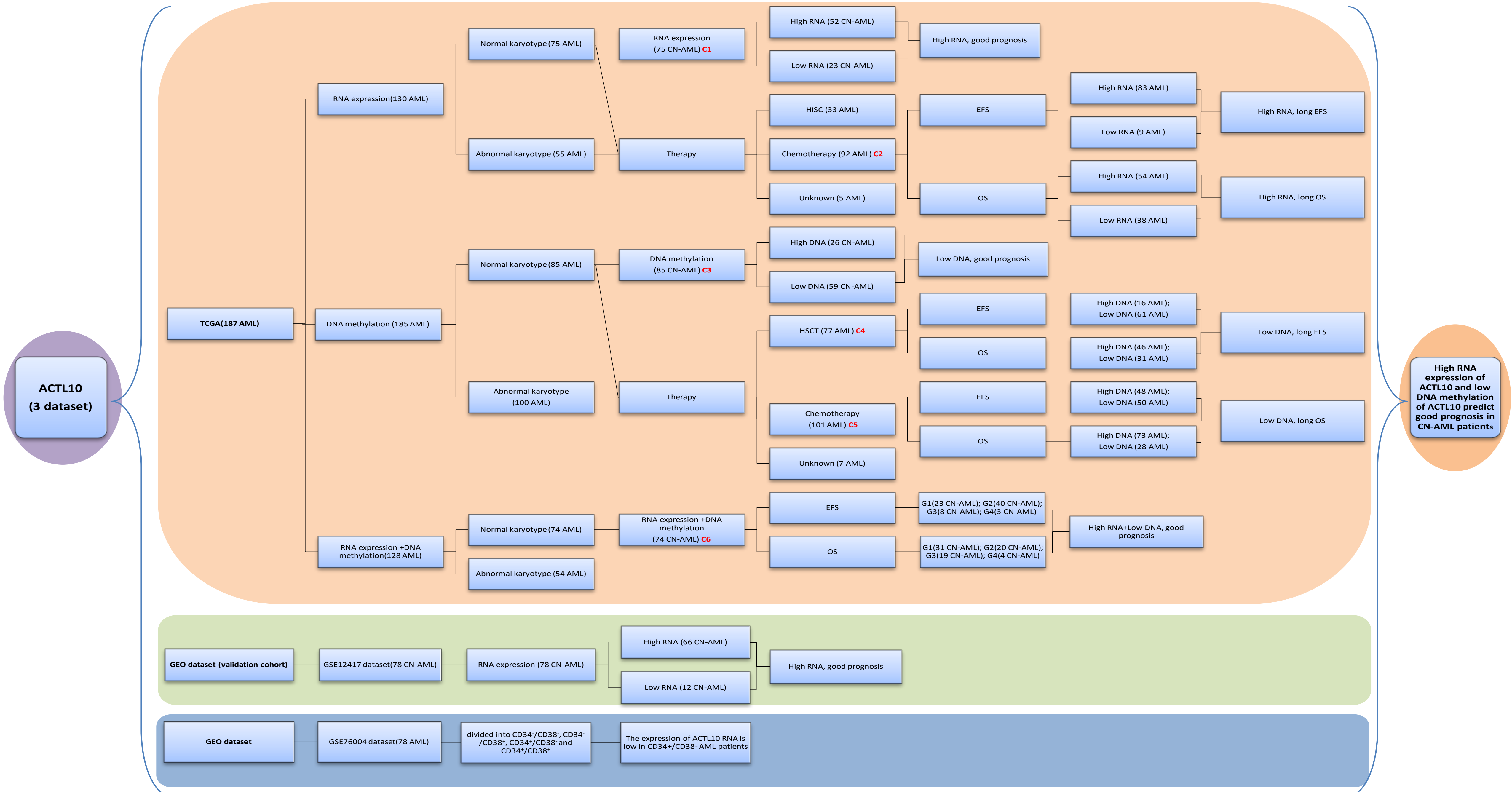

Supplementary Figure 2

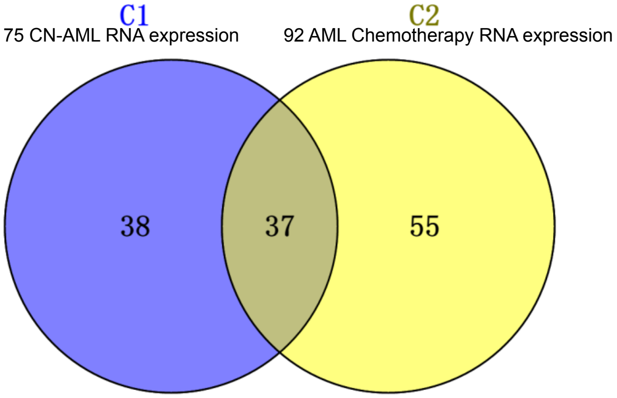

A: Overlap of patients with RNA expression

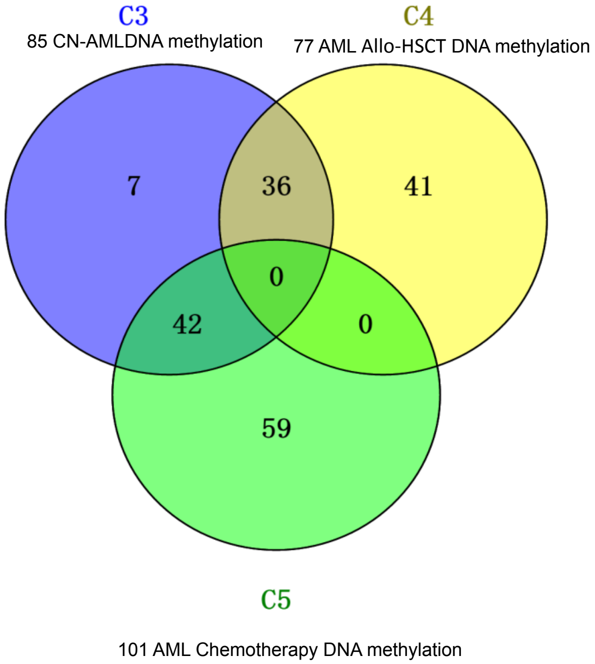

B: Overlap of DNA methylation patients

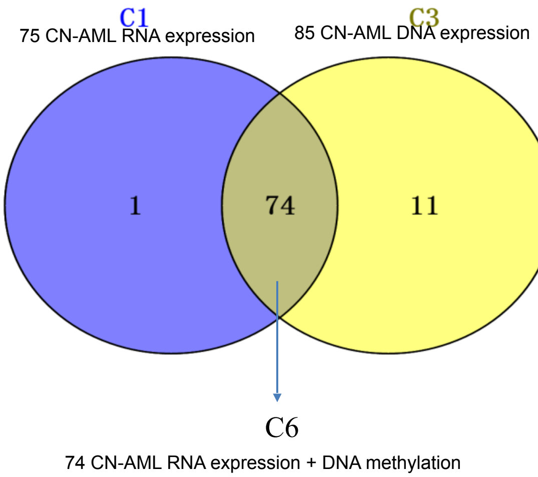

C: Overlap of CN-AML patients

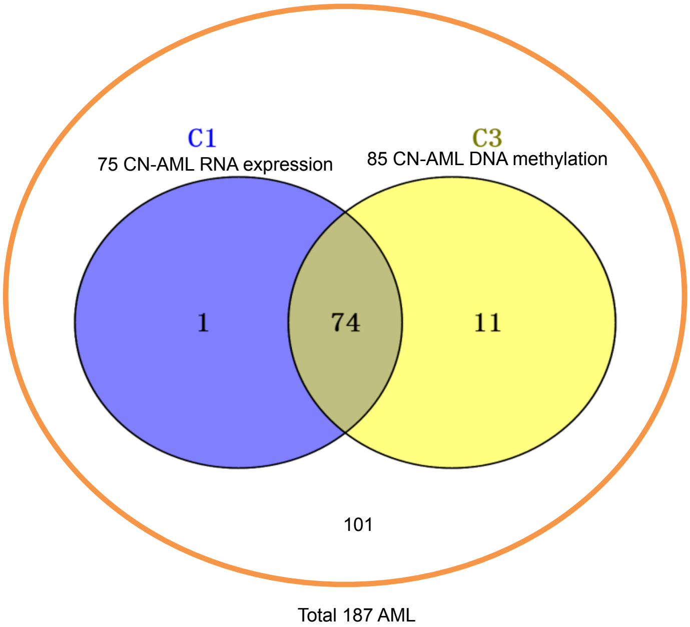

D: The proportion of CN-AML patients in all patients

Supplementary Figure 3

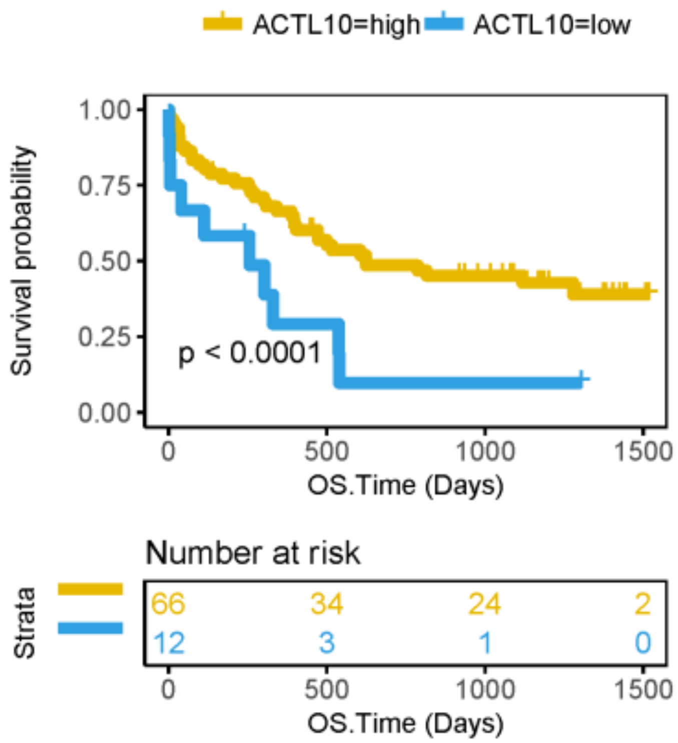

Supplementary Figure 4

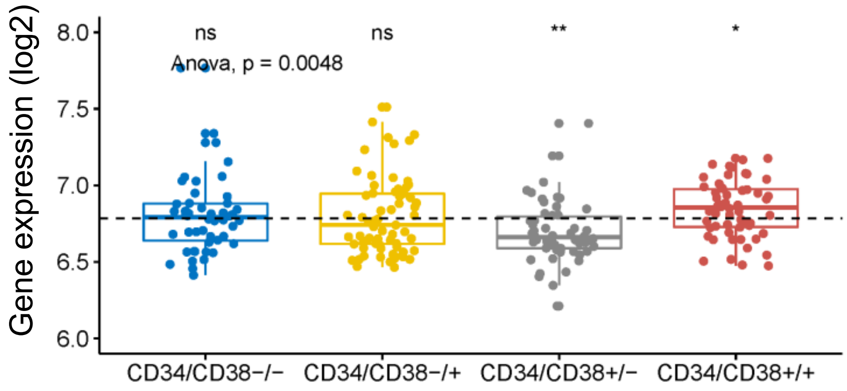

Supplement Table 1: Information of all patients participating in each groups in the TCGA dataset.

Number	Sample_ID	Karyotype	RNA-seq	DNA-methy	Therapy	Participating group				
**1**	TCGA-AB-2805-03	Normal	Yes	Yes	Chemotherapy	C1	C2	C3	C5	C6
**2**	TCGA-AB-2811-03	Normal	Yes	Yes	Chemotherapy	C1	C2	C3	C5	C6
**3**	TCGA-AB-2816-03	Normal	Yes	Yes	Chemotherapy	C1	C2	C3	C5	C6
**4**	TCGA-AB-2818-03	Normal	Yes	Yes	Chemotherapy	C1	C2	C3	C5	C6
**5**	TCGA-AB-2824-03	Normal	Yes	Yes	Chemotherapy	C1	C2	C3	C5	C6
**6**	TCGA-AB-2826-03	Normal	Yes	Yes	Chemotherapy	C1	C2	C3	C5	C6
**7**	TCGA-AB-2833-03	Normal	Yes	Yes	Chemotherapy	C1	C2	C3	C5	C6
**8**	TCGA-AB-2835-03	Normal	Yes	Yes	Chemotherapy	C1	C2	C3	C5	C6
**9**	TCGA-AB-2836-03	Normal	Yes	Yes	Chemotherapy	C1	C2	C3	C5	C6
**10**	TCGA-AB-2837-03	Normal	Yes	Yes	Chemotherapy	C1	C2	C3	C5	C6
**11**	TCGA-AB-2851-03	Normal	Yes	Yes	Chemotherapy	C1	C2	C3	C5	C6
**12**	TCGA-AB-2853-03	Normal	Yes	Yes	Chemotherapy	C1	C2	C3	C5	C6
**13**	TCGA-AB-2866-03	Normal	Yes	Yes	Chemotherapy	C1	C2	C3	C5	C6
**14**	TCGA-AB-2867-03	Normal	Yes	Yes	Chemotherapy	C1	C2	C3	C5	C6
**15**	TCGA-AB-2869-03	Normal	Yes	Yes	Chemotherapy	C1	C2	C3	C5	C6
**16**	TCGA-AB-2873-03	Normal	Yes	Yes	Chemotherapy	C1	C2	C3	C5	C6
**17**	TCGA-AB-2879-03	Normal	Yes	Yes	Chemotherapy	C1	C2	C3	C5	C6
**18**	TCGA-AB-2900-03	Normal	Yes	Yes	Chemotherapy	C1	C2	C3	C5	C6
**19**	TCGA-AB-2903-03	Normal	Yes	Yes	Chemotherapy	C1	C2	C3	C5	C6
**20**	TCGA-AB-2919-03	Normal	Yes	Yes	Chemotherapy	C1	C2	C3	C5	C6
**21**	TCGA-AB-2925-03	Normal	Yes	Yes	Chemotherapy	C1	C2	C3	C5	C6
**22**	TCGA-AB-2927-03	Normal	Yes	Yes	Chemotherapy	C1	C2	C3	C5	C6
**23**	TCGA-AB-2932-03	Normal	Yes	Yes	Chemotherapy	C1	C2	C3	C5	C6

**24**	TCGA-AB-2938-03	Normal	Yes	Yes	Chemotherapy	C1	C2	C3	C5	C6
**25**	TCGA-AB-2948-03	Normal	Yes	Yes	Chemotherapy	C1	C2	C3	C5	C6
**26**	TCGA-AB-2964-03	Normal	Yes	Yes	Chemotherapy	C1	C2	C3	C5	C6
**27**	TCGA-AB-2971-03	Normal	Yes	Yes	Chemotherapy	C1	C2	C3	C5	C6
**28**	TCGA-AB-2972-03	Normal	Yes	Yes	Chemotherapy	C1	C2	C3	C5	C6
**29**	TCGA-AB-2973-03	Normal	Yes	Yes	Chemotherapy	C1	C2	C3	C5	C6
**30**	TCGA-AB-2977-03	Normal	Yes	Yes	Chemotherapy	C1	C2	C3	C5	C6
**31**	TCGA-AB-2984-03	Normal	Yes	Yes	Chemotherapy	C1	C2	C3	C5	C6
**32**	TCGA-AB-2987-03	Normal	Yes	Yes	Chemotherapy	C1	C2	C3	C5	C6
**33**	TCGA-AB-2988-03	Normal	Yes	Yes	Chemotherapy	C1	C2	C3	C5	C6
**34**	TCGA-AB-2996-03	Normal	Yes	Yes	Chemotherapy	C1	C2	C3	C5	C6
**35**	TCGA-AB-3002-03	Normal	Yes	Yes	Chemotherapy	C1	C2	C3	C5	C6
**36**	TCGA-AB-3006-03	Normal	Yes	Yes	Chemotherapy	C1	C2	C3	C5	C6
**37**	TCGA-AB-2812-03	Normal	Yes	Yes	HTSC	C1	C3	C4	C6	
**38**	TCGA-AB-2839-03	Normal	Yes	Yes	HTSC	C1	C3	C4	C6	
**39**	TCGA-AB-2848-03	Normal	Yes	Yes	HTSC	C1	C3	C4	C6	
**40**	TCGA-AB-2871-03	Normal	Yes	Yes	HTSC	C1	C3	C4	C6	
**41**	TCGA-AB-2877-03	Normal	Yes	Yes	HTSC	C1	C3	C4	C6	
**42**	TCGA-AB-2880-03	Normal	Yes	Yes	HTSC	C1	C3	C4	C6	
**43**	TCGA-AB-2884-03	Normal	Yes	Yes	HTSC	C1	C3	C4	C6	
**44**	TCGA-AB-2896-03	Normal	Yes	Yes	HTSC	C1	C3	C4	C6	
**45**	TCGA-AB-2909-03	Normal	Yes	Yes	HTSC	C1	C3	C4	C6	
**46**	TCGA-AB-2921-03	Normal	Yes	Yes	HTSC	C1	C3	C4	C6	
**47**	TCGA-AB-2924-03	Normal	Yes	Yes	HTSC	C1	C3	C4	C6	
**48**	TCGA-AB-2934-03	Normal	Yes	Yes	HTSC	C1	C3	C4	C6	
**49**	TCGA-AB-2936-03	Normal	Yes	Yes	HTSC	C1	C3	C4	C6	

**50**	TCGA-AB-2955-03	Normal	Yes	Yes	HTSC	C1	C3	C4	C6
**51**	TCGA-AB-2963-03	Normal	Yes	Yes	HTSC	C1	C3	C4	C6
**52**	TCGA-AB-2965-03	Normal	Yes	Yes	HTSC	C1	C3	C4	C6
**53**	TCGA-AB-2966-03	Normal	Yes	Yes	HTSC	C1	C3	C4	C6
**54**	TCGA-AB-2969-03	Normal	Yes	Yes	HTSC	C1	C3	C4	C6
**55**	TCGA-AB-2970-03	Normal	Yes	Yes	HTSC	C1	C3	C4	C6
**56**	TCGA-AB-2975-03	Normal	Yes	Yes	HTSC	C1	C3	C4	C6
**57**	TCGA-AB-2976-03	Normal	Yes	Yes	HTSC	C1	C3	C4	C6
**58**	TCGA-AB-2978-03	Normal	Yes	Yes	HTSC	C1	C3	C4	C6
**59**	TCGA-AB-2981-03	Normal	Yes	Yes	HTSC	C1	C3	C4	C6
**60**	TCGA-AB-2983-03	Normal	Yes	Yes	HTSC	C1	C3	C4	C6
**61**	TCGA-AB-2986-03	Normal	Yes	Yes	HTSC	C1	C3	C4	C6
**62**	TCGA-AB-2990-03	Normal	Yes	Yes	HTSC	C1	C3	C4	C6
**63**	TCGA-AB-2992-03	Normal	Yes	Yes	HTSC	C1	C3	C4	C6
**64**	TCGA-AB-2993-03	Normal	Yes	Yes	HTSC	C1	C3	C4	C6
**65**	TCGA-AB-2995-03	Normal	Yes	Yes	HTSC	C1	C3	C4	C6
**66**	TCGA-AB-3000-03	Normal	Yes	Yes	HTSC	C1	C3	C4	C6
**67**	TCGA-AB-3008-03	Normal	Yes	Yes	HTSC	C1	C3	C4	C6
**68**	TCGA-AB-3009-03	Normal	Yes	Yes	HTSC	C1	C3	C4	C6
**69**	TCGA-AB-3011-03	Normal	Yes	Yes	HTSC	C1	C3	C4	C6
**70**	TCGA-AB-2825-03	Normal	Yes	Yes	unknown	C1	C3	C6	
**71**	TCGA-AB-2845-03	Normal	Yes	Yes	unknown	C1	C3	C6	
**72**	TCGA-AB-2931-03	Normal	Yes	Yes	unknown	C1	C3	C6	
**73**	TCGA-AB-2967-03	Normal	Yes	Yes	unknown	C1	C3	C6	
**74**	TCGA-AB-2979-03	Normal	Yes	Yes	unknown	C1	C3	C6	
**75**	TCGA-AB-2859-03	Normal	Yes		Chemotherapy	C1	C2		

**76**	TCGA-AB-2803-03	t(15;17)/PML-RARA	Yes	Yes	Chemotherapy	C2	C5
**77**	TCGA-AB-2807-03	21	Yes	Yes	Chemotherapy	C2	C5
**78**	TCGA-AB-2813-03	Complex	Yes	Yes	Chemotherapy	C2	C5
**79**	TCGA-AB-2819-03	t(8;21)/RUNX1-RUNX1T1	Yes	Yes	Chemotherapy	C2	C5
**80**	TCGA-AB-2820-03	Complex	Yes	Yes	Chemotherapy	C2	C5
**81**	TCGA-AB-2823-03	t(15;17)/PML-RARA	Yes	Yes	Chemotherapy	C2	C5
**82**	TCGA-AB-2828-03	inv(16)/CBFB-MYH11	Yes	Yes	Chemotherapy	C2	C5
**83**	TCGA-AB-2830-03	Others	Yes	Yes	Chemotherapy	C2	C5
**84**	TCGA-AB-2832-03	11q23/MLL trans	Yes	Yes	Chemotherapy	C2	C5
**85**	TCGA-AB-2834-03	11q23/MLL trans	Yes	Yes	Chemotherapy	C2	C5
**86**	TCGA-AB-2838-03	Complex	Yes	Yes	Chemotherapy	C2	C5
**87**	TCGA-AB-2840-03	t(15;17)/PML-RARA	Yes	Yes	Chemotherapy	C2	C5
**88**	TCGA-AB-2841-03	t(15;17)/PML-RARA	Yes	Yes	Chemotherapy	C2	C5
**89**	TCGA-AB-2843-03	Others	Yes	Yes	Chemotherapy	C2	C5
**90**	TCGA-AB-2844-03	11q23/MLL trans	Yes	Yes	Chemotherapy	C2	C5
**91**	TCGA-AB-2856-03	11p15/NUP98 trans	Yes	Yes	Chemotherapy	C2	C5
**92**	TCGA-AB-2858-03	t(8;21)/RUNX1-RUNX1T1	Yes	Yes	Chemotherapy	C2	C5
**93**	TCGA-AB-2861-03	8	Yes	Yes	Chemotherapy	C2	C5
**94**	TCGA-AB-2862-03	t(15;17)/PML-RARA	Yes	Yes	Chemotherapy	C2	C5
**95**	TCGA-AB-2863-03	8	Yes	Yes	Chemotherapy	C2	C5
**96**	TCGA-AB-2872-03	t(15;17)/PML-RARA	Yes	Yes	Chemotherapy	C2	C5
**97**	TCGA-AB-2875-03	t(8;21)/RUNX1-RUNX1T1	Yes	Yes	Chemotherapy	C2	C5
**98**	TCGA-AB-2882-03	-7	Yes	Yes	Chemotherapy	C2	C5
**99**	TCGA-AB-2885-03	Complex	Yes	Yes	Chemotherapy	C2	C5
**100**	TCGA-AB-2886-03	t(8;21)/RUNX1-RUNX1T1	Yes	Yes	Chemotherapy	C2	C5
**101**	TCGA-AB-2888-03	inv(16)/CBFB-MYH11	Yes	Yes	Chemotherapy	C2	C5

**102**	TCGA-AB-2890-03	Others	Yes	Yes	Chemotherapy	C2	C5
**103**	TCGA-AB-2895-03	Unknow	Yes	Yes	Chemotherapy	C2	C5
**104**	TCGA-AB-2897-03	t(15;17)/PML-RARA	Yes	Yes	Chemotherapy	C2	C5
**105**	TCGA-AB-2899-03	Others	Yes	Yes	Chemotherapy	C2	C5
**106**	TCGA-AB-2908-03	Complex	Yes	Yes	Chemotherapy	C2	C5
**107**	TCGA-AB-2914-03	inv(16)/CBFB-MYH11	Yes	Yes	Chemotherapy	C2	C5
**108**	TCGA-AB-2915-03	Complex	Yes	Yes	Chemotherapy	C2	C5
**109**	TCGA-AB-2928-03	-7	Yes	Yes	Chemotherapy	C2	C5
**110**	TCGA-AB-2929-03	Complex	Yes	Yes	Chemotherapy	C2	C5
**111**	TCGA-AB-2933-03	Others	Yes	Yes	Chemotherapy	C2	C5
**112**	TCGA-AB-2935-03	Complex	Yes	Yes	Chemotherapy	C2	C5
**113**	TCGA-AB-2937-03	t(8;21)/RUNX1-RUNX1T1	Yes	Yes	Chemotherapy	C2	C5
**114**	TCGA-AB-2942-03	inv(16)/CBFB-MYH11	Yes	Yes	Chemotherapy	C2	C5
**115**	TCGA-AB-2943-03	Complex	Yes	Yes	Chemotherapy	C2	C5
**116**	TCGA-AB-2944-03	-7	Yes	Yes	Chemotherapy	C2	C5
**117**	TCGA-AB-2950-03	t(8;21)/RUNX1-RUNX1T1	Yes	Yes	Chemotherapy	C2	C5
**118**	TCGA-AB-2952-03	Complex	Yes	Yes	Chemotherapy	C2	C5
**119**	TCGA-AB-2954-03	inv(16)/CBFB-MYH11	Yes	Yes	Chemotherapy	C2	C5
**120**	TCGA-AB-2956-03	11q23/MLL trans	Yes	Yes	Chemotherapy	C2	C5
**121**	TCGA-AB-2959-03	Others	Yes	Yes	Chemotherapy	C2	C5
**122**	TCGA-AB-2980-03	t(15;17)/PML-RARA	Yes	Yes	Chemotherapy	C2	C5
**123**	TCGA-AB-2985-03	11q23/MLL trans	Yes	Yes	Chemotherapy	C2	C5
**124**	TCGA-AB-2991-03	t(15;17)/PML-RARA	Yes	Yes	Chemotherapy	C2	C5
**125**	TCGA-AB-2998-03	t(15;17)/PML-RARA	Yes	Yes	Chemotherapy	C2	C5
**126**	TCGA-AB-2999-03	t(15;17)/PML-RARA	Yes	Yes	Chemotherapy	C2	C5
**127**	TCGA-AB-3001-03	t(15;17)/PML-RARA	Yes	Yes	Chemotherapy	C2	C5

**128**	TCGA-AB-3007-03	t(15;17)/PML-RARA	Yes	Yes	Chemotherapy	C2	C5
**129**	TCGA-AB-3012-03	t(15;17)/PML-RARA	Yes	Yes	Chemotherapy	C2	C5
**130**	TCGA-AB-2827-03	Normal		Yes	HTSC	C3	C4
**131**	TCGA-AB-2907-03	Normal		Yes	HTSC	C3	C4
**132**	TCGA-AB-2989-03	Normal		Yes	HTSC	C3	C4
**133**	TCGA-AB-2802-03	Normal		Yes	Chemotherapy	C3	C5
**134**	TCGA-AB-2809-03	Normal		Yes	Chemotherapy	C3	C5
**135**	TCGA-AB-2923-03	Normal		Yes	Chemotherapy	C3	C5
**136**	TCGA-AB-2947-03	Normal		Yes	Chemotherapy	C3	C5
**137**	TCGA-AB-2968-03	Normal		Yes	Chemotherapy	C3	C5
**138**	TCGA-AB-2974-03	Normal		Yes	Chemotherapy	C3	C5
**139**	TCGA-AB-2865-03	21	Yes		Chemotherapy	C2	
**140**	TCGA-AB-2831-03	Normal		Yes	unknown	C3	
**141**	TCGA-AB-2922-03	Normal		Yes	unknown	C3	
**142**	TCGA-AB-2804-03	t(15;17)/PML-RARA		Yes	HTSC	C4	
**143**	TCGA-AB-2806-03	t(8;21)/RUNX1-RUNX1T1		Yes	HTSC	C4	
**144**	TCGA-AB-2808-03	Others		Yes	HTSC	C4	
**145**	TCGA-AB-2814-03	Others		Yes	HTSC	C4	
**146**	TCGA-AB-2815-03	inv(16)/CBFB-MYH11		Yes	HTSC	C4	
**147**	TCGA-AB-2817-03	t(9;22)/BCR-ABL1		Yes	HTSC	C4	
**148**	TCGA-AB-2821-03	8		Yes	HTSC	C4	
**149**	TCGA-AB-2822-03	8		Yes	HTSC	C4	
**150**	TCGA-AB-2846-03	inv(16)/CBFB-MYH11		Yes	HTSC	C4	
**151**	TCGA-AB-2847-03	Others		Yes	HTSC	C4	
**152**	TCGA-AB-2849-03	Complex		Yes	HTSC	C4	
**153**	TCGA-AB-2855-03	Complex		Yes	HTSC	C4	

**154**	TCGA-AB-2857-03	Complex	Yes	HTSC	C4
**155**	TCGA-AB-2860-03	Complex	Yes	HTSC	C4
**156**	TCGA-AB-2874-03	-7	Yes	HTSC	C4
**157**	TCGA-AB-2876-03	Others	Yes	HTSC	C4
**158**	TCGA-AB-2878-03	Complex	Yes	HTSC	C4
**159**	TCGA-AB-2881-03	nv(16)/CBFB-MYH11	Yes	HTSC	C4
**160**	TCGA-AB-2883-03	11q23/MLL trans	Yes	HTSC	C4
**161**	TCGA-AB-2887-03	Others	Yes	HTSC	C4
**162**	TCGA-AB-2889-03	inv(16)/CBFB-MYH11	Yes	HTSC	C4
**163**	TCGA-AB-2891-03	Complex	Yes	HTSC	C4
**164**	TCGA-AB-2892-03	nv(16)/CBFB-MYH11	Yes	HTSC	C4
**165**	TCGA-AB-2893-03	11q23/MLL trans	Yes	HTSC	C4
**166**	TCGA-AB-2898-03	8	Yes	HTSC	C4
**167**	TCGA-AB-2901-03	t(9;22)/BCR-ABL1	Yes	HTSC	C4
**168**	TCGA-AB-2904-03	Complex	Yes	HTSC	C4
**169**	TCGA-AB-2911-03	11q23/MLL trans	Yes	HTSC	C4
**170**	TCGA-AB-2912-03	8	Yes	HTSC	C4
**171**	TCGA-AB-2913-03	8	Yes	HTSC	C4
**172**	TCGA-AB-2916-03	Others	Yes	HTSC	C4
**173**	TCGA-AB-2917-03	Complex	Yes	HTSC	C4
**174**	TCGA-AB-2918-03	11p15/NUP98 trans	Yes	HTSC	C4
**175**	TCGA-AB-2920-03	Complex	Yes	HTSC	C4
**176**	TCGA-AB-2930-03	11p15/NUP98 trans	Yes	HTSC	C4
**177**	TCGA-AB-2939-03	Complex	Yes	HTSC	C4
**178**	TCGA-AB-2940-03	21	Yes	HTSC	C4
**179**	TCGA-AB-2945-03	Complex	Yes	HTSC	C4

**180**	TCGA-AB-2946-03	Others	Yes	HTSC	C4
**181**	TCGA-AB-2949-03	Complex	Yes	HTSC	C4
**182**	TCGA-AB-2997-03	t(15;17)/PML-RARA	Yes	HTSC	C4
**183**	TCGA-AB-2829-03	Others	Yes	Chemotherapy	C5
**184**	TCGA-AB-2870-03	inv(16)/CBFB-MYH11	Yes	Chemotherapy	C5
**185**	TCGA-AB-2906-03	t(15;17)/PML-RARA	Yes	Chemotherapy	C5
**186**	TCGA-AB-2957-03	inv(16)/CBFB-MYH11	Yes	Chemotherapy	C5
**187**	TCGA-AB-2982-03	t(15;17)/PML-RARA	Yes	Chemotherapy	C5

Note: RNA-seq: Patients with RNA sequencing data. DNA-methy: Patients with DNA methylation data. HSCT: Patients receiving Allo-HSCT. C1: 75 CN-AML patients who compared survival time (EFS and OS) between ACTL10 RNA-high expression group and ACTL10 RNA-low expression group. C2: 92 AML patients received chemotherapy who compared clinical prognosis between ACTL10 RNA-high expression group and ACTL10 RNA-low expression group. C3: 85 CN-AML patients who compared clinical prognosis between ACTL10 DNA methylation-high group and the ACTL10 DNA methylation-low group. C4: 77 AML patients received Allo-HSCT who compared clinical prognosis between ACTL10 DNA methylation-high group and the ACTL10 DNA methylation-low group. C5: 101 AML patients received chemotherapy who compared clinical prognosis between ACTL10 DNA methylation-high group and the ACTL10 DNA methylation-low group. C6: 74 CN-AML who integrated ACTL10 RNA expression data with ACTL10 DNA methylation data.
